# Spatial heterogeneity of cancer associated protein expression in immunohistochemically stained images as an improved prognostic biomarker

**DOI:** 10.3389/fonc.2022.964716

**Published:** 2022-12-19

**Authors:** Henrik Failmezger, Harald Hessel, Ansh Kapil, Günter Schmidt, Nathalie Harder

**Affiliations:** Research & Development Early Oncology Translational Medicine, Computational Pathology, AstraZeneca Computational Pathology GmbH, Munich, Germany

**Keywords:** spatial heterogeneity, immunohistochemistry, digital pathology, spatial statistics, prognostic biomarker

## Abstract

The identification of new tumor biomarkers for patient stratification before therapy, for monitoring of disease progression, and for characterization of tumor biology plays a crucial role in cancer research. The status of these biomarkers is mostly scored manually by a pathologist and such scores typically, do not consider the spatial heterogeneity of the protein’s expression in the tissue. Using advanced image analysis methods, marker expression can be determined quantitatively with high accuracy and reproducibility on a per-cell level. To aggregate such per-cell marker expressions on a patient level, the expression values for single cells are usually averaged for the whole tissue. However, averaging neglects the spatial heterogeneity of the marker expression in the tissue. We present two novel approaches for quantitative scoring of spatial marker expression heterogeneity. The first approach is based on a co-occurrence analysis of the marker expression in neighboring cells. The second approach accounts for the local variability of the protein’s expression by tiling the tissue with a regular grid and assigning local spatial heterogeneity phenotypes per tile. We apply our novel scores to quantify the spatial expression of four different membrane markers, i.e., HER2, CMET, CD44, and EGFR in immunohistochemically (IHC) stained tissue sections of colorectal cancer patients. We evaluate the prognostic relevance of our spatial scores in this cohort and show that the spatial heterogeneity scores clearly outperform the marker expression average as a prognostic factor (CMET: p-value=0.01 vs. p-value=0.3).

## Introduction

In cancer research, tumor biomarkers play a crucial role for patient stratification in targeted therapies, for monitoring of disease progression, and for characterization of tumor biology. For example HER2 expression is an important biomarker for immunotherapies in breast cancer (1, 2). Applying advanced image analysis and machine learning methods, the quantitative expression of IHC markers can be determined for whole tissue sections with high accuracy. Such methods have been shown to be highly concordant to the manual scoring of a marker by pathologists (3, 4) and provide quantitative measurements of the marker’s expression on a cellular level. To aggregate the cellular expression values into one score per sample, most often simple averaging is performed which does not consider the spatial heterogeneity of the marker expression (5). However, it has been shown that relevant prognostic information can be derived by analyzing the spatial composition of the tumor microenvironment (6–8), as well as more generally the spatial heterogeneity of protein expression. For example, intra-tumoral heterogeneity has been found to be associated with more aggressive tumors and unfavorable outcomes in many cancer indications (9).

To assess heterogeneity in marker expression, Cherenova et al. have transferred cell signal intensity levels of immunofluorescence images to marks in marked point patterns and defined scores based on the conditional mean and variance of this point pattern (5). They showed that their scores provide prognostic information in breast cancer.

In this technical proof of concept study, we present spatial scores to determine the expression heterogeneity of proteins in the tissue which turned out to have enormous potential to serve as future biomarkers in clinical studies.

In comparison to Cherenova et al. our approach does not view the tissue as a marked point pattern but is inspired by the well-known Haralick features (10) that are used for texture analysis in pixel images. Like the Haralick features, our scores are based on a co-occurrence analysis, however instead of analyzing grey value intensities of neighboring pixels, we examine the marker expressions of neighboring cells. Our method has the advantage that it can be applied to continuous measurements like marker expression intensities and that it enables the calculation of a variety of scores that capture different aspects of marker expression heterogeneity.

As the tumor microenvironment is highly complex and marker expression may vary locally in the tissue, we implemented a second methodology that is based on a tessellation of the tumor tissue. By overlaying the tissue with a grid, we quantify the local distribution of marker expression. The cells inside the grid tiles are classified into *marker-positive* and *marker-negative* by a range of intensity thresholds. Based on the *marker-positive* and *marker-negative* cell proportions, we calculate the Shannon entropy as a measure of local heterogeneity. We then consider different approaches to aggregate the local heterogeneity values into global per-sample scores.

We apply our novel scoring approaches to resections from a cohort of 34 colorectal cancer patients that have been stained by immunohistochemistry for HER2, CMET, CD44, and EGFR. We show that the spatial scores provide a considerable improvement in patient stratification as compared to the standard averaging of the marker values.

## Methods

### Data

**Figure 1:**
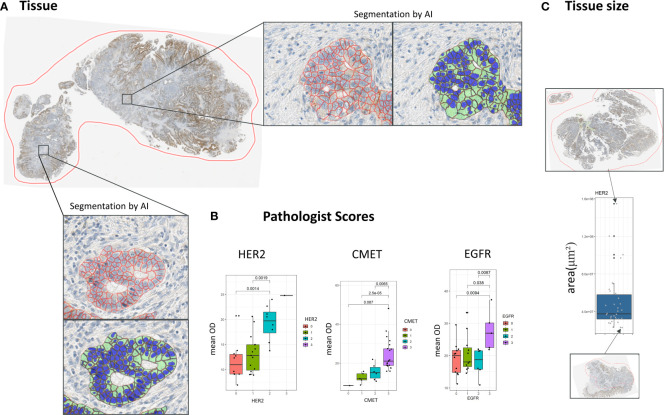
Cell segmentation pipeline. **(A)** The membrane of cells is segmented by an automated segmentation pipeline. **(B)** Concordance of scores resulting from the automatic image analysis pipeline with pathologist scores. **(C)** Distribution of tissue sizes in the dataset in *μ*m^2^.

The cohort included 34 patients of colorectal cancer (CRC). FFPE blocks (Indivumed) of tumor resections were stained for the membrane markers EGFR (3C6), HER2 (4B5), CMET (ECD), and CD44 (**Figure 1C**). Samples were scanned by Aperio with a magnification of 20x.

There were 34 samples available for each CMET, HER2, and EGFR, and 31 samples for CD44. All patients had late stage tumors (Stage IV = 33 patients, Stage II = 1 patient), 74% of the patients were treated by chemotherapy, for 15% therapy was unknown (**Supplementary Figure S1**). A subset of patients (41%) was also treated by targeted therapy (Cetuximab (Erbitux®)/Bevacizumab (Avastin®)). Overall survival data was available for all patients.

### Image segmentation

In all whole slide tissue images epithelium was detected automatically and all epithelial cells were segmented into three compartments (nucleus, cytoplasm, membrane) using a dedicated image analysis approach described in more detail elsewhere (11). In short, the approach is based on two independent supervised convolutional neural network (CNN) models for semantic segmentation. The first model robustly detects epithelium regions while the second model predicts the cellular compartments for the whole image. In a postprocessing step the predictions of both CNNs are combined to compute the cell segmentation within the detected epithelium regions (**Figure 1A**). Both models have been trained on large amounts of IHC stained tissue images along with expert annotations including different indications and markers. Finally, based on the segmentation result the amount of brown DAB staining on the cellular membranes of all tumor cells was quantified by computing the average brown optical density (mean OD) across all pixels per cellular membrane. Note that tumor epithelium (vs. normal epithelium) was classified based on coarse tumor core region annotations provided by an expert pathologist. The performance of the segmentation approach on the images of this study has been assessed by correlating the image-based readouts with scoring performed by expert pathologists (**Figure 1B**).

### Co-occurrence scores

**Figure 2:**
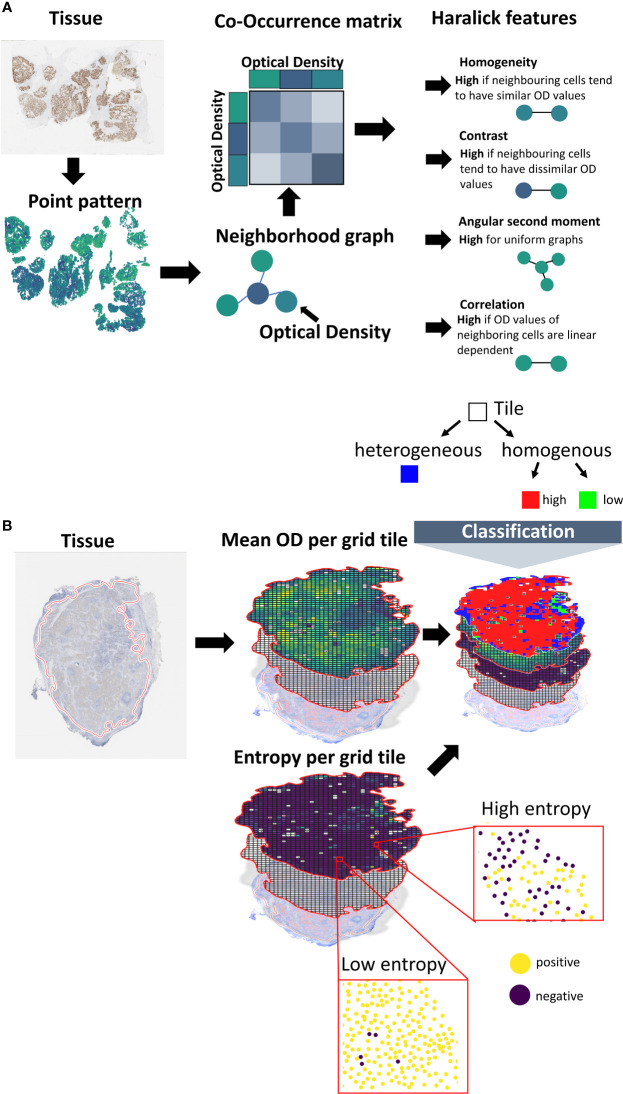
Calculation of spatial scores. **(A)** Spatial scores based on co-occurrence analysis. Using a machine learning pipeline, the cells are transferred into a point pattern. Center coordinates and marker membrane OD values are reported per cell. The point pattern is transformed into a neighborhood graph. Cells correspond to nodes and edges connect cells in spatial proximity (using a distance criterion). A co-occurrence matrix is created, where the entries of the matrix represent the frequencies in OD value combinations for pairs of neighboring cells. Scores are calculated from the co-occurrence matrix based on the Haralick texture features. **(B)** Tessellation scores. The tissue is overlaid with a grid. The averaged membrane OD values of the cells that fall inside a certain tile are calculated. Cells are divided into *marker-positive* and *marker-negative* based on a threshold. The Shannon entropy is calculated for the class proportions. Tiles are classified into *marker-homogenous* or *-heterogenous* based on the Shannon entropy. Homogenous tiles are classified into *marker-high* or *marker-low* based on a threshold.

The co-occurrence scores are inspired by the Haralick texture analysis (10). In comparison to the Haralick features, we do not create a pixel-based but an object-based co-occurrence matrix (12), where objects correspond to cells in the tissue. To this end, we first create a cell neighborhood graph, in which cells are considered as nodes and edges represent the neighborhood relation of cells based on a distance threshold (**Figure 2A**). Based on this cell graph a co-occurrence matrix is calculated as a 2D histogram of membrane optical density values for all pairs of cells sharing an edge in the neighborhood graph. The size of the resulting co-occurrence matrix is given by 255x255, which corresponds to the mapped OD values. Based on the co-occurrence matrix, we calculate a subset of the original aralick features, including *Homogeneity*, *Contrast*, *Correlation*, *Angular second moment* (10).

Let *p*(*i*,*j*) be the normalized co-occurrence matrix, with *i,j* being the OD values of the cells. Let *μ*_*x*_, *μ*_*y*_ and  *σ*_*x*_, *σ*_*y*_  be the marginal means and standard deviations of the rows or columns of the co-occurrence matrix. $Homogeneity=\sum_{i}\sum_{j}\frac{1}{1+({i-j)}^{2}}p\left(i,j\right)$ is large if neighboring cells share similar OD values, whereas $Contrast=\sum_{i}\sum_{j}{\left(i-j\right)}^{2}p\left(i,j\right)$ increases if neighboring cells have distinct OD values. $Correlation=\frac{\sum_{i}\sum_{\text{j}}(ij)p(i,j)-{µ}_{x}{µ}_{y}}{\sigma_{x}\sigma_{y}}$ is closely related to the Pearson correlation statistic and measures the linear dependency of the expression values of neighboring cells. $Angular\;second\;moment\;=\sum_{i}\sum_{j}p{\left(i,j\right)}^{2}$ is a measure of general uniformity of OD values.

As the co-occurrence matrix is dependent on the choice of the distance threshold, we created separate co-occurrence matrices for a range of distances (10, 25, 50, 75 μm), resulting in 16 co-occurrence scores per tissue sample.

### Tessellation scores

The second set of scores is based on tissue tessellation. We first calculate the relevant tissue region by the convex hull of all cell coordinates. This region is split into subregions based on a grid with a region size of 250x250 μm². Only tiles that include at least n=5 cells are considered for further analysis. The class *high expression* or *low expression* is assigned based on the marker expression of the cells (i.e., membrane mean OD) inside the grid tile using a fixed threshold. From the resulting spatial expression map (**Figure 2B**) a single score is calculated as the ratio of the expression classes. To make this score more independent from the localization of the tiles, and thus, robust against tiling artefacts, we shift the grid by 25 μm in all 4 directions. The resulting score is the average of the scores of the individual shifts.

As an extension to the binary expression classes, we also assess the expression heterogeneity per tile. Therefore, we first classify the cells into *marker-positive* or *marker-negative* cells by a pre-defined threshold on the membrane mean OD values. We then calculate the Shannon entropy of the class proportions per tile. The per-tile entropy values are aggregated into a single score by taking their average and by calculating the so called, Ecosystem Diversity Index (7) (EDI). The EDI represents the number of components within the distribution of the per-tile entropy values. These components can be seen as the number of sub-populations in cellular expression heterogeneity. To identify the components, a range of Gaussian mixture models with different component numbers (1–5) are fitted to the distribution. The number of components of the best fitting Gaussian mixture model (w.r.t. Bayesian information criterion) defines the EDI of a sample (**Supplementary Figure S2**). Additionally, we calculate the EDI of all mean expression values and of the standard deviation of the expression values.

Next, we also incorporate the class *heterogenous* into the spatial expression map (**Figure 2**). Therefore, we apply a decision tree-like scheme, by first classifying cells inside the tile into *homogenous* or *heterogenous* expression based on the Shannon entropy values (**Figures 2**; **S2**). For this classification we use an entropy threshold of 0.61 that corresponds to maximum proportions of 70% positive and 30% negative cells (or vice versa). Tiles below this value are then classified as *homogenous-high* or *homogenous-low*. For the classification into *homogenous-high* or *homogenous-low*, we use the OD thresholds [8, 10, 15, 20, 25]. The corresponding range of threshold is used to calculate the Shannon entropy by classifying the individual cells into *marker-positive* vs. *marker-negative* as described above.

### Feature selection and analysis

To reduce the overall number of scores, and thus avoid overfitting, spatial scores with a Pearson correlation larger than 0.9 were pruned. In the correlation filtering process, we applied univariate Cox regression to select the scores to keep, i.e. for a set of correlated scores we kept the score that had the best association with survival (i.e., lowest p-value). We then applied feature selection by *lasso* (least absolute shrinkage and selection operator) regression to the remaining set of uncorrelated scores using a stability selection approach (13). Only scores that had a selection probability of 0.20 in the stability selection were used for further analysis.

We used univariate and multiple Cox regression to determine the relationship of the scores with overall survival. In order to avoid multicolinearity in the multiple regression approach, we used only scores that had a Spearman correlation smaller than 0.9 with the average marker expression. We reduced the covariates by stepwise regression analysis using forward-backward selection (14).

### Cross validation

To evaluate the robustness of the selected spatial scores we implemented an n-times repeated k-fold cross validation scheme. Therefore, we first implemented a k-fold cross validation scheme, in which the full data was partitioned into k equally-sized random splits. For a given split, all but one of the k-subsets were used for training an optimal cutoff. The cutoffs were then applied to the remaining unseen subset to test stratification performance. To evaluate log-rank performance, high and low scoring patients were grouped across all k test folds within one split to derive a cross-validated Kaplan-Meier curves. To test the statistical significance of the Kaplan-Meier curves, a permutation test (15) was applied to derive empirical p-values using m random permutations of the overall survival information. The above-described k-fold cross validation was then applied to each of the permutations to derive the distribution of the log-rank statistic under the Null hypothesis of independence of considered features and outcome. We used the parameters n=20, k=10, m=1000.

### Software and implementation

The image analysis pipeline was implemented in Python and Tensorflow (16). The calculation of the spatial scores was implemented in Python. *Scikit-learn* was used for fitting the Gaussian mixture model (17), *scikit-image* was used to calculate the Haralick features.

Data analysis was conducted in R. The package *survminer* was used for the survival analysis. The package *lmtest* was used for the likelihood ratio test (18). The *stabs* package (19) was used for the stability selection approach. The package *MASS* was used for stepwise regression analysis (14).

## Results

### Mapping IHC marker expression to spatial scores

To calculate spatial heterogeneity scores for the expression of the four considered markers HER2, CMET, CD44, and EGFR the images were first processed by a machine learning pipeline that included region segmentation, cell segmentation and cell classification (11). We compared the results of the image analysis pipeline with pathologist scores for HER2, CMET, and EGFR (note that no pathologist scores were available for CD44) and found an overall good concordance between the average marker expression and *high/low* pathologist scores (**Figure 1B**).

**Figure 3:**
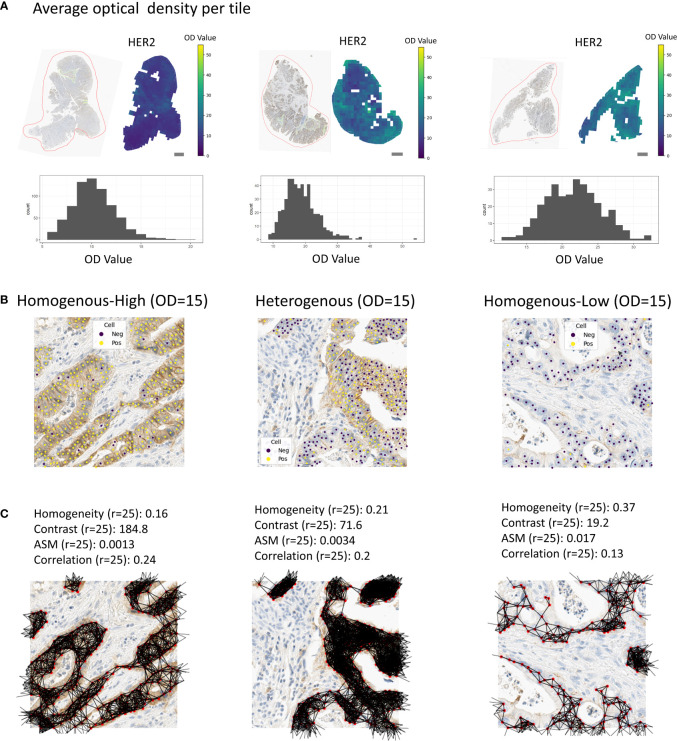
Tissue regions with associated spatial scores. **(A)** Examples of tissue samples with associated tile-wise optical densities (HER2). Scale bars correspond to *1mm*. **(B)** Examples of homogenous-high, heterogenous or homogenous-low tiles. **(C)** Associated co-occurrence features. Co-occurrence features were calculated on the cells in the tiles only.

We calculated four different co-occurrence scores based on the Haralick features for texture analysis: *Homogeneity*, *Contrast*, *Angular second moment (ASM)* and *Correlation* (**Figures 2**; **3C**, Methods). To calculate co-occurrence scores for different ranges, we used the radii of 10, 25, 50, and 75 μm to define cellular neighborhood in the neighborhood graph construction (**Figure 3C**). For a radius of 10 µm we found cells to have a median of 2.3 neighbors, whereas for a radius of 75 µm cells had a median of 109.3 neighbors (**Figure S3**). We further found the *Homogeneity* as well as the *Correlation* feature to be larger for small radii and decreasing for larger radii, whereas the feature values for the other features stayed stable for different radii (**Supplementary Figure S4**).

For the tessellation scores, we overlaid the tissue with a quadratic grid of tile size 250x250 μm². We observed a large variety in per-tile marker expression over the whole tissue (**Figure 3A**). In order to classify these tiles into the categories *homogenous-high* and *homogenous-low* (see Methods, **Figure 3B**), we used the OD thresholds of 8, 10, 15, 20, and 25. We used the same range of thresholds to calculate the entropy of *marker-positive* or *marker-negative* cells (Methods). We calculated the Ecosystem Diversity Index (EDI) for the average marker expression per tile and the standard deviation of this marker expression (**Supplementary Figure S2**). Furthermore, we calculated the EDI for the entropy of *marker-positive* or *marker-negative* cell proportions, resulting in 7 EDI scores for one sample as well as ratios for the numbers of *homogenous-high*, *homogenous-low*, and *heterogenous* tiles (**Figure 3B**). To assess the robustness of our tessellation scores with respect to the size and positioning of the tiles on the tissue we performed additional experiments. To account for the dependency of the scores on the tile location, we shifted the grid in all directions by 1/10 of the tile size (i.e., 25 µm) and computed the average score over all shifts as the final readout. To investigate the dependency of the tessellation scores on the tile size we increased and decreased the tile size by 50%, respectively, and compared the resulting scores for one feature. We found a very good concordance between the scores calculated on different tile sizes (Pearson correlation > 99%, **Supplementary Figure S5**) suggesting a solid robustness with respect to the choice of this parameter.

In total we computed 16 co-occurrence scores and 37 tessellation scores. We found high correlations between the tesselation scores and the co-occurrence scores. In general, scores based on *Correlation* and EDI were less correlated with the rest of the scores (**Supplementary Figure S6**).

To select the most relevant subset of spatial scores based on their prognostic value we first pruned correlated scores, and afterwards applied Cox lasso stability selection (see Methods). We kept all scores that were selected in at least 20% of the stability selection runs. From the initial set of 53 spatial scores, 5 scores were selected for HER2, 4 scores for CMET, 4 scores for CD44, and 4 scores for EGFR (**Table S1**). For the selected spatial scores, we also checked the correlation with pathologist scores. We found only 5 spatial scores to be clearly correlated with pathologist scores (HER2: *Ratio of homogenous-low tiles* (OD=8); *Ratio of homogenous-high tiles* (OD=25); CMET: *Homogeneity* (r=75), Contrast (OD=10), *EDI for the entropy* (OD=20), **Supplementary Figure S7**), indicating that the spatial scores provide additional information, which is not represented by pathologist scores.

### Univariate analysis

**Figure 4:**
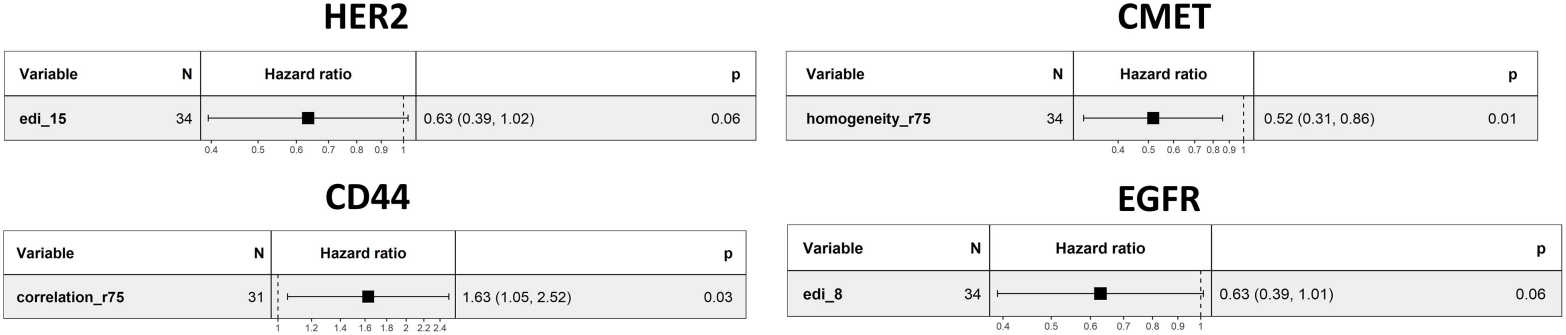
Univariate regression analysis for different markers. The scores with the lowest p-values are shown.

We checked if the selected spatial scores had an association with overall survival by univariate Cox regression analysis. *Homogeneity* (r=75, p-value=0.01, **Figure 4**) was associated with survival for CMET, whereas large *Homogeneity* reduced the patient’s Hazard (HR=0.52). For CD44 *Correlation* (r=75) was significantly associated with survival and increased the patient’s Hazard (p-value=0.03, HR=1.63, **Figure 4**). We did not find any other significant univariate associations of the spatial scores with overall survival. We additionally applied univariate cox regression for the average marker expression values, but also found no significant association to overall survival (**Supplementary Figure S8**).

### Multiple regression analysis

We checked if the spatial scores provide additional prognostic value compared to simple aggregation of the expression values by global averaging.

Therefore, we created baseline multiple regression models including the averaged marker expression values as well as the clinical variables age, gender, tumor grading, and the pathologist score for the marker expression (**Supplementary Figure S9**). We added the spatial scores selected by lasso regression and reduced the model by stepwise regression. We then compared the advanced model to the base model by a Likelihood-Ratio test to check whether the spatial scores provided additional prognostic value.

**Figure 5:**
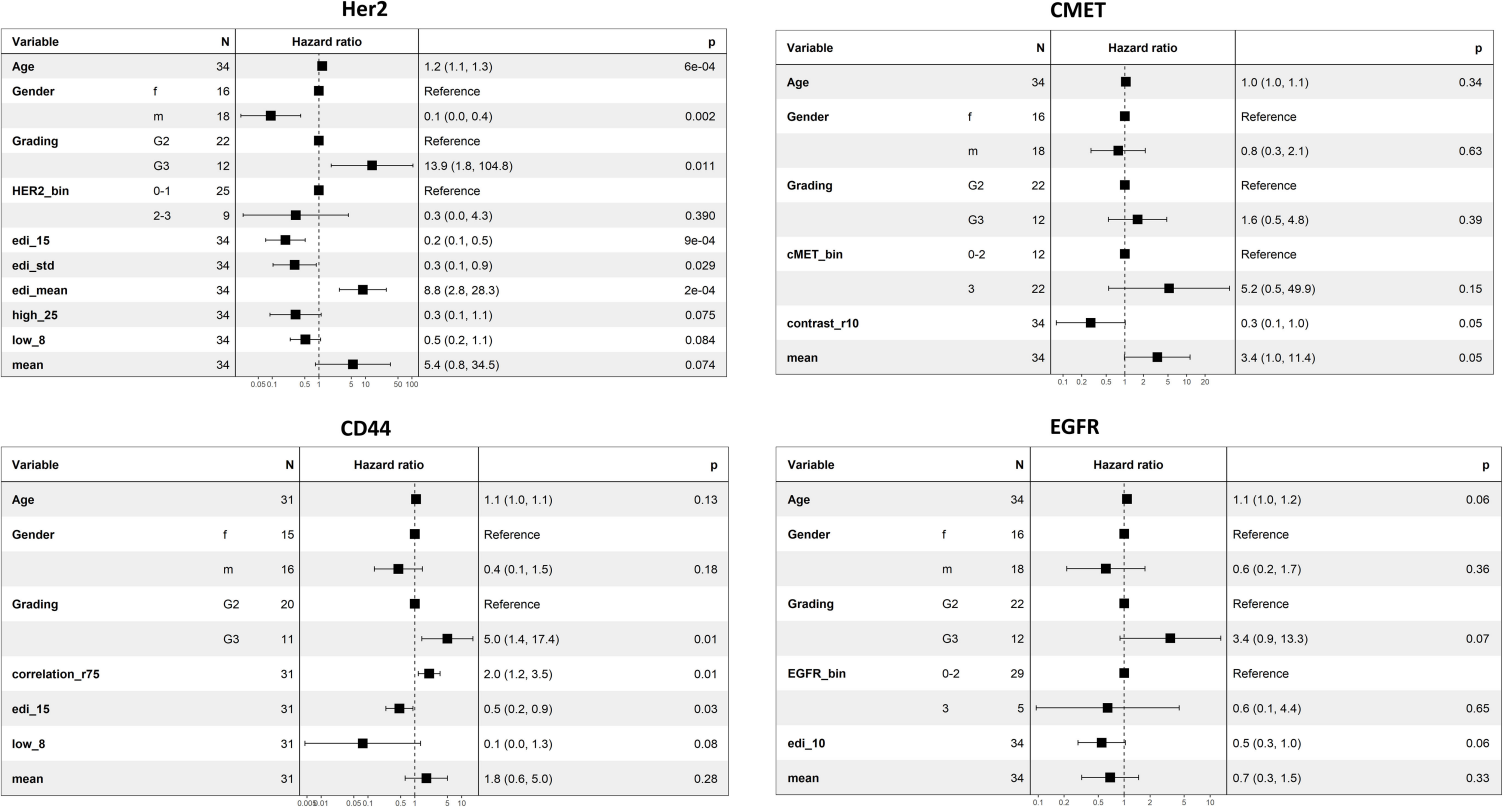
Multiple regression analysis for the markers HER2, CMET, CD44, and EGFR. Spatial scores were added to a base model including age, gender, grading, pathologist scoring, and average marker expression, and afterwards reduced by stepwise regression analysis. The improvement of the advanced model to the base model was calculated by the Likelihood-Ratio test (HER2: p-value = 2.909e-06, CMET: p-value= 0.018, CD44: p-value=0.003, EGFR: p-value=0.066). Note that pathologist scores were not available for CD44.

For HER2, the ratio of *homogenous-high* tiles (OD=25) and *homogenous-low* tiles (OD=8), the *EDI* for the entropy (OD=15), the *EDI* for the average expression, and the *EDI* for the standard deviation in expression (Methods) remained in the model after stepwise selection and added significant prognostic value to the baseline model (LR-test: p-value= 2.909e-06, **Figure 5**). For CMET, *Contrast* (r=10, **Figure 5**) was kept in the model and significantly improved its prognostic value (LR-test: p-value= 0.018, **Figure 5**). For CD44, *Correlation* (r=75), the *EDI* for the entropy (OD=15) and the ratio of *homogenous-low* tiles (OD=8), remained in the model (LR-test: p-value=0.003, **Figure 5**). For EGFR only the *EDI* for the entropy (OD=10) was kept in the model (LR-test: p-value=0.066, **Figure 5**).

### Patient stratification

**Figure 6:**
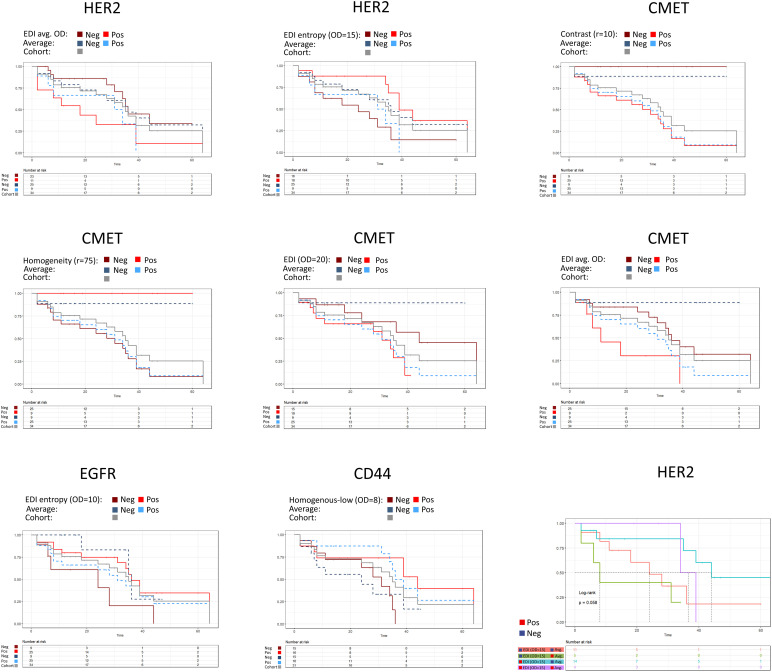
Patient stratification by spatial scores. The average expression is shown as dashed line. 25%, 50% or 75% quantiles of the score were used as stratification cutoffs, respectively, and the best cutoff was selected based on the p-value of the logrank test. HER2: *EDI* (average OD per tile, 75% quantile, p-value=0.04), *EDI* (entropy, OD=15, 50% quantile, p-value=0.017), average expression (75% quantile, p-value=0.23). CMET: *Contrast* (r=10, 75% quantile, p-value=0.002), Homogeneity (r=75, 75% quantile, p-value=0.002), *EDI* (OD=20, 50% quantile, p-value=0.049), *EDI* (average OD per tile, 75% quantile, p-value=0.023), average expression (25% quantile, p-value=0.015). EGFR: *EDI* (entropy, OD=10, 25% quantile, p-value=0.046), average expression (25% quantile, p-value=0.28). CD44: *Ratio of homogenous-low tiles* (OD=8, 50% quantile, p-value=0.025), average expression (50% quantile, p-value=0.07). In the bivariate analysis scores were grouped by the cutoff from the univariate analysis.

To test if the spatial scores can be used to stratify patients by overall survival, we tested the 25%, 50%, and 75% quantiles, respectively, as potential cutoffs for patient selection. We created survival curves for the patient groups and compared the difference of survival times by the logrank test. We found 8 features to be significant in the logrank test for the different markers. The *EDI* for the averaged OD per tile (75% quantile, p-value=0.04, **Figure 6**) and the *EDI* for the entropy (OD=15, 50% quantile, p-value=0.017, **Figure 6**) were significant for HER2. The *Contrast* (r=10, 75% quantile, p-value=0.002), the *Homogeneity* (r=75, 75% quantile, p-value=0.002, **Figure 6**), the *EDI* for the entropy (OD=20, 50% quantile, p-value=0.049), and *EDI* for the averaged OD per tile (average OD per tile, 75% quantile, p-value=0.023) were significant for CMET. Whereas only one score each were significant for CD44 (*ratio of homogenous-low tiles* (OD=8, 50% quantile, p-value=0.025, **Figure 6**), and for EGFR (*EDI* for the entropy, OD=10, 25% quantile, p-value=0.046, **Figure 6**). We found the average marker expression to be significant only in CMET (p-value=0.015, **Figure 6**).

We checked the robustness of our scores by a 20 x 10 repeated cross validation scheme (Methods). Three spatial scores had a significant median p-value in the repeated cross validation scheme (**Supplementary Figure S10**, CMET: *Homogeneity* (r=75), median p-value=0.033; *Contrast* (r=10), median p-value=0.009; *EDI* for the averaged OD, median p-value=0.043).

As the spatial scores do not indicate if the marker expression in the tissue in general is high or low, we combined the spatial scores with the average marker expression. We then grouped patients based on the categories high or low obtained for both feature values using the cutoff from the univariate analysis. For HER2, patients with low average expression and high *EDI* (entropy, OD=15) had the best prognosis (median survival time), whereas patients with low *EDI* (entropy, OD=15) and high average expression had the worst prognosis (**Figure 6**). We found similar group stratifications for EGFR, CMET and CD44 (**Supplementary Figure S11**).

## Discussion

Aggregating cellular information from tissue slides is a challenging problem and applying straightforward methods such as simple averaging leads to a massive loss of information. The tumor microenvironment is a highly complex system, which can either trigger or downregulate protein expression. Finding appropriate scores that adequately capture the marker’s expression heterogeneity in the tissue is crucial to better characterize and identify different patient populations.

We presented two novel scoring approaches that aggregate cellular protein expression values to per-sample scores while quantifying the spatial heterogeneity in protein expression. Both approaches turned out to have immense potential for use as prognostic and potentially predictive biomarkers for patient stratification.

Our scoring approaches are widely applicable as they are generally independent of imaging modality or indication, they can be applied on any type of continuous measurements, and they can easily be adjusted to a different domain with an extremely small set of free parameters to be tuned. For the co-occurrence scores the only parameter to be specified is the distance criterion for setting up the cell neighborhood graph. The tessellation scores depend on three parameters that are (1) the tile size (2), the threshold on the continuous per-cell measurement to classify a tile as *homogenous-high* or *low* (in our case the membrane OD threshold), and (3) the threshold to classify a tile as *homogenous* or *heterogenous*. In our study we only varied parameter (2) the membrane OD threshold to compute a set of spatial scores corresponding to different resolutions of spatial dependency, and we used fixed values for the two other parameters. Finding an appropriate grid size for spatial analysis tasks in digital pathology is non-trivial and there exists no general solution (6, 7, 20). However, in our study we systematically investigated the influence of the tile size on the overall result, and we showed that our changes of 50% of the tile size had no relevant effect on the results (**Supplementary Figure S5**). However, larger variations of the tile sizes could certainly be employed to capture heterogeneity at different scales. The threshold to classify homogenous versus heterogenous tiles was fixed to an entropy value of 0.61, meaning that tiles were classified as heterogenous if less than 70% of the cells were either positive or negative. We did not optimize this value to avoid overfitting, however, adaptation of this value might be reasonable, for example, for other cancer indications.

In order to evaluate the generality of the scores, we tested them on a cohort of CRC patients for four different markers with known distinct expression patterns (21–23): HER2, CD44, CMET, and EGFR.

All four markers are well known to be associated with unfavorable prognosis in CRC (24–27). We applied a Cox lasso stability feature selection scheme and observed that tessellation scores as well as co-occurrence scores were selected as most relevant with respect to patient stratification for different markers (**Supplementary Table S1**). The spatial scores showed a higher prognostic value than the average cellular expression, underlining the importance of the spatial heterogeneity of the cellular expression values. This finding was confirmed by univariate and multiple cox regression. Overall, the spatial scores generally stratified patients better than averaging.

Interestingly, some scores were only found significant when analyzed together with the average expression in the multiple regression model (**Figure 5**). This potentially is due to suppression effects. Suppression effects in multiple regression happen, if a predictor (suppressor) that is uncorrelated with the output criterion, but is correlated with another predictor, improves the overall predictive power of a model (28). The reason for that is that the suppressor controls for variance in the other predictor. For the spatial scores this indicates a general challenge. The spatial scores identify spatially *homogenous* or *heterogenous* expression of the marker, however, they provide no information about the absolute value of the marker expression - meaning they do not indicate whether the marker is strongly or weakly expressed in the tissue. The combination of our spatial scores with the average expression considerably improved the prognostic value of the multiple regression model suggesting great potential for patient stratification. We showed this, for example, for HER2 where the combination of the spatial scores with the average expression resulted in a refined patient stratification into four groups with quite distinct prognosis (**Figure 6**).

The main limitation of this technical proof of concept study is the small dataset. In future work, the significance of our scores needs to be reproduced in a larger cohort, ideally including genomic and proteomic data.

## Data availability statement

The original contributions presented in the study are included in the article/**Supplementary Material**. Further inquiries can be directed to the corresponding author.

## Ethics statement

Samples used in this study were provided by Individumed GmbH, 20251 Hamburg, Germany. Written agreements for all relevant samples were signed, guaranteeing that appropriate ethics approvals were obtained. The patients/participants provided their written informed consent to participate in this study.

## Author contributions

HF developed the scores, implemented the methods, analyzed the data, and wrote the manuscript. NH contributed to the development of the scores, to the image analysis and manuscript revision. AK contributed to the image analysis pipeline and provided technical advice. HH stained the tumor sections. GS provided the data and contributed to the development of the scores. All authors contributed to the article and approved the submitted version.
