# Supplementary material for: Spatial heterogeneity of cancer associated protein expression in immunohistochemically stained images as an improved prognostic biomarker
Source: Front Oncol. 2022 Dec 19;12:964716. doi: 10.3389/fonc.2022.964716 (PMC9806230; doi:10.3389/fonc.2022.964716)
Supplement: Supplementary file 1 [file DataSheet_1.pdf]

Supplementary Material

Spatial heterogeneity of cancer associated protein expression in immunohistochemically stained images as an improved prognostic biomarker

**Characteristic**	**CRC**, N = 34[1]
gender	
female	16 (47%)
male	18 (53%)
primary.or.relapse.tumor	
primary tumor	34 (100%)
chemotherapy	
no	4 (12%)
unknown	5 (15%)
yes	25 (74%)
targeted.therapies	
no	15 (44%)
unknown	5 (15%)
yes	14 (41%)

[1] n (%)

Figure S1 Data description.

Her2	CMET	CD44	EGFR
edi_15	contrast_r10	correlation_r75	edi_8
edi_std	homogeneity_r75	edi_mean	edi_10
edi_mean	edi_mean	edi_15	edi_20
low_8	edi_20	low_8	heterogenous_20
high_25			

Table S1 Selected features resulting from Cox lasso stability selection.

EDI

In order to calculate the Ecosystem diversity index, cells are first binarized by a threshold into positive and negative cells. The threshold corresponds to the threshold that is also used to classify a tile into homogenous-high or homogenous-low (Methods).

The Shannon entropy is calculated for the percentages of positive and negative cells per tile. High entropy values correspond to rather equivalent proportions of positive and negative cells and thus to a higher heterogeneity. In order to calculate the *EDI*, Gaussian Mixture Models (GMMs) with different component numbers (1-5) are fitted to the distribution of tile entropy values. We select the best Gaussian Mixture Model based on the Bayesian Information Criterion (BIC). The BIC is a criterion for model selection which chooses the best model based on its likelihood whereas at the same time penalizes the number of parameters. The GMM with the smallest BIC was selected. The number of components of the best fitting GMM then corresponds to the *EDI*.

We also calculate the EDI for the average OD per tile and the standard deviation of the OD in a tile. In this case the distribution of entropy values is replaced by the distribution of average OD values per tile or by the standard deviation of OD values per tile, respectively.

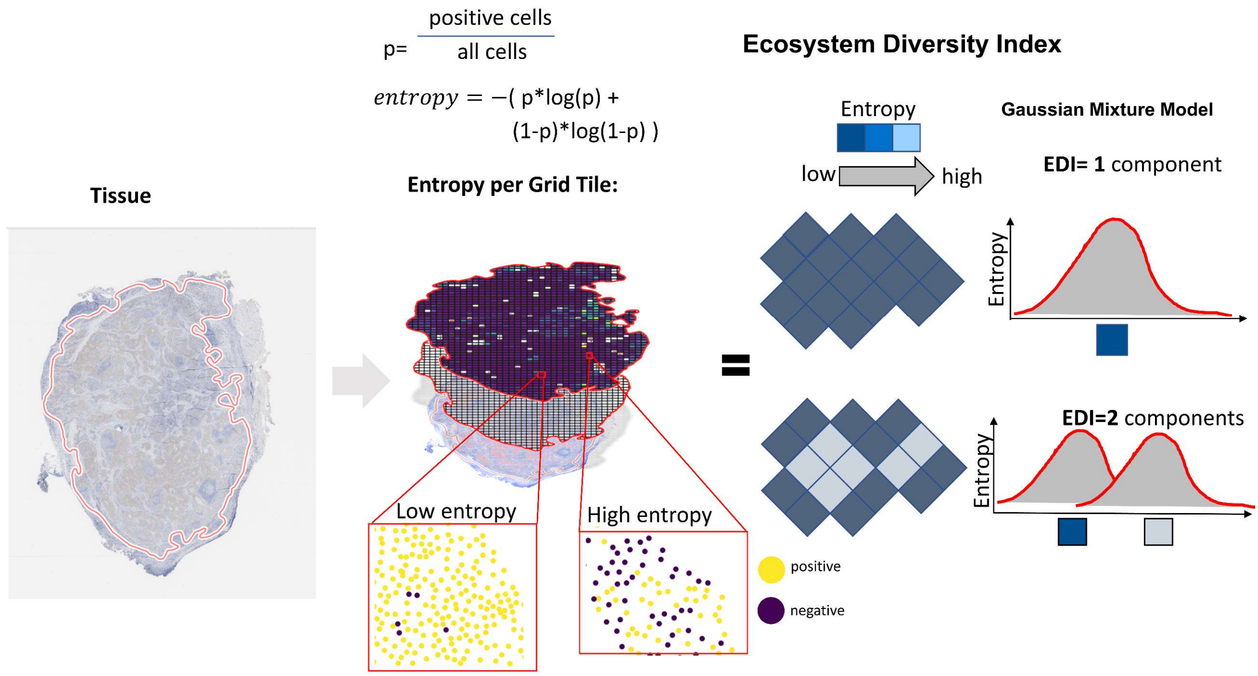

Figure S2 Calculation of the EDI.

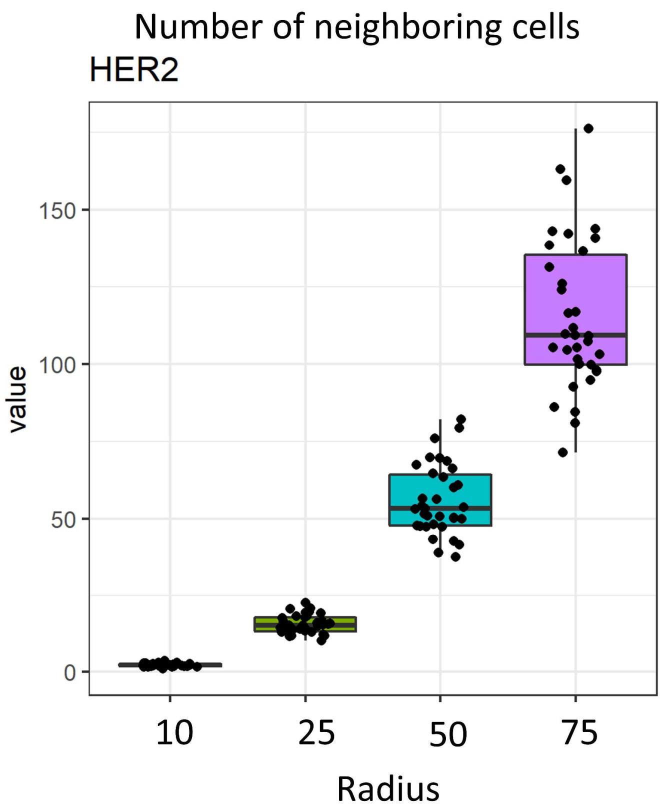

Figure S3 Average number of neighbors for different distance radii (radius given in μm).

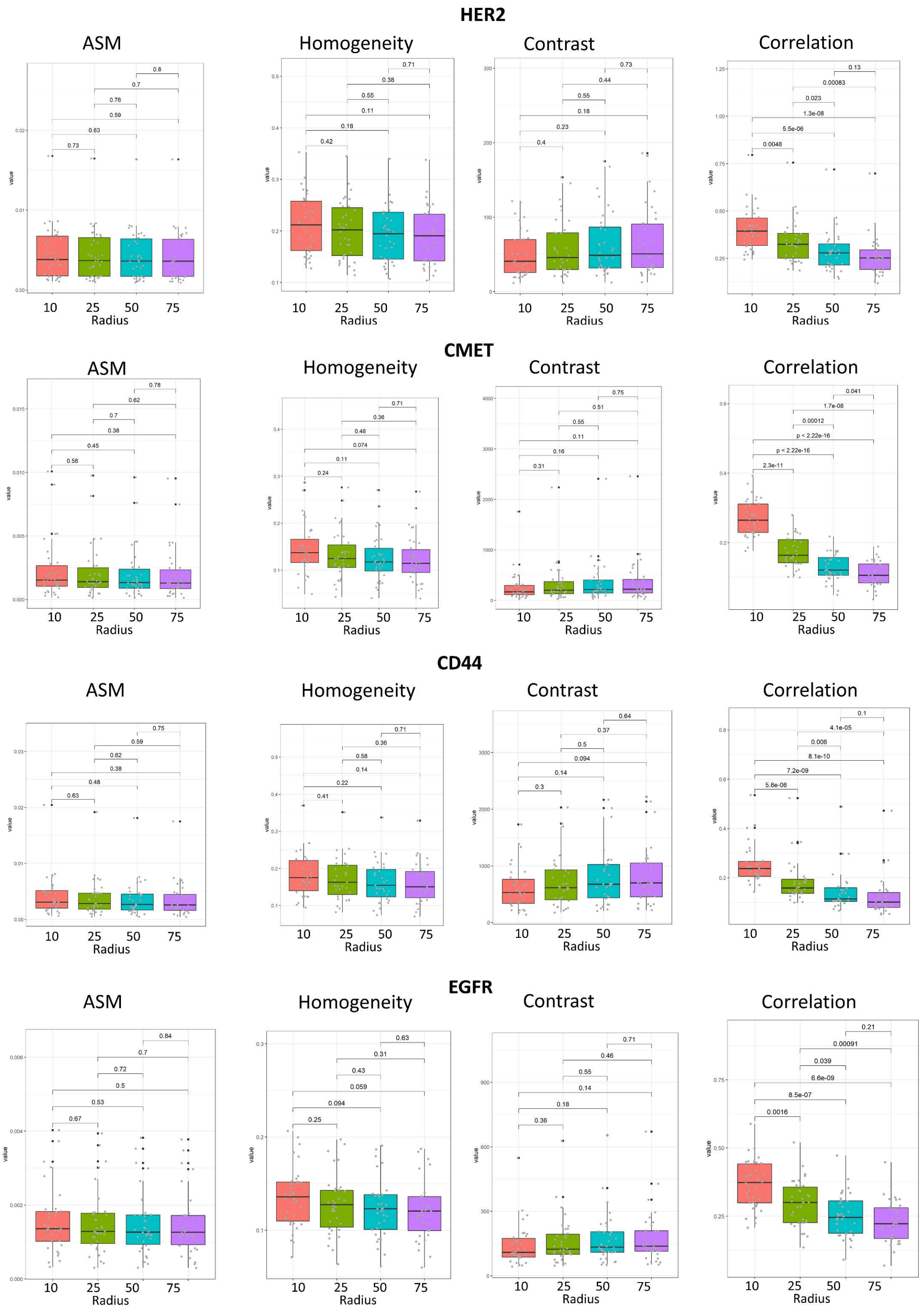

Figure S4 Values of co-occurrence features for different distance radii. Radii given in µm.

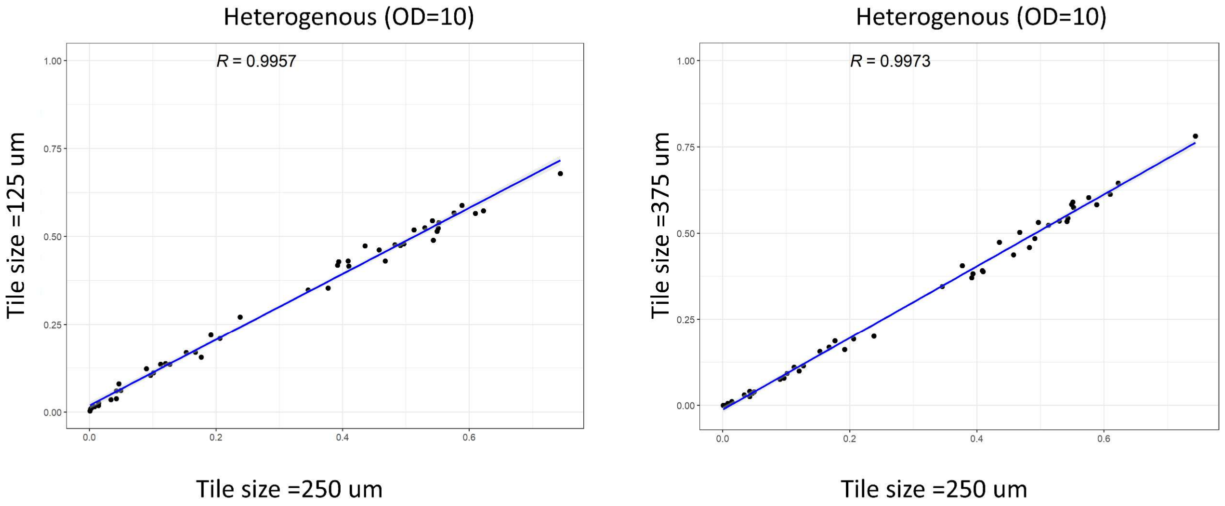

Figure S5 Feature comparison for different tile sizes.

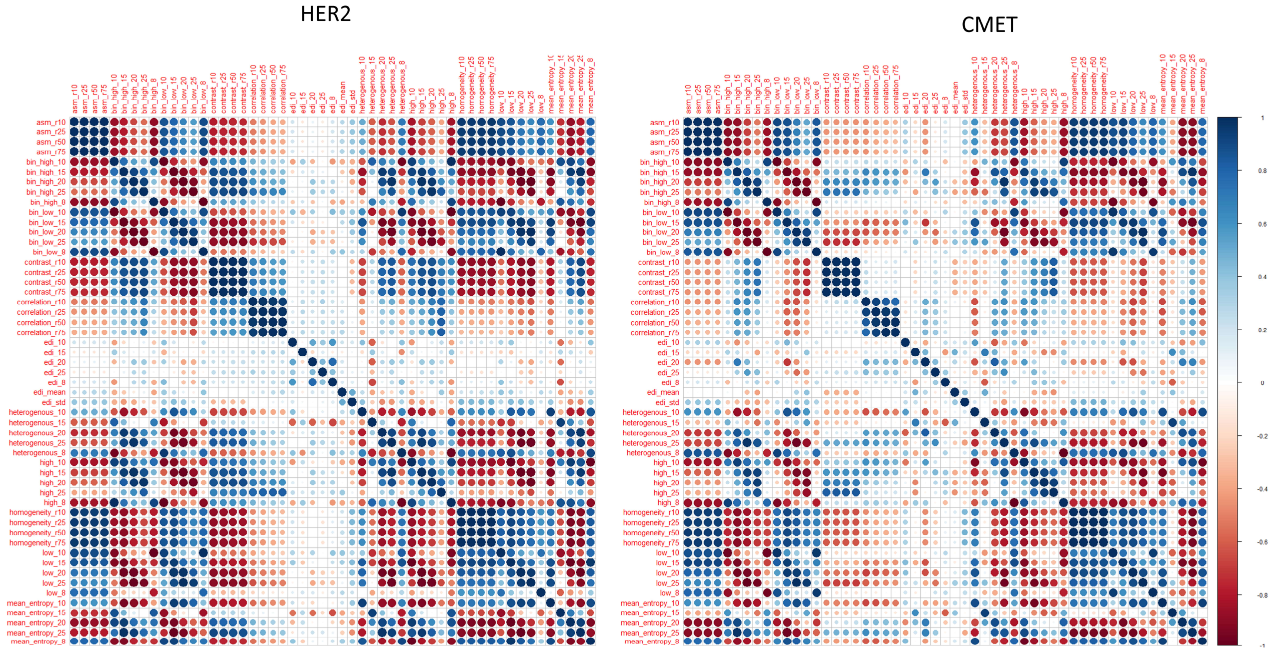

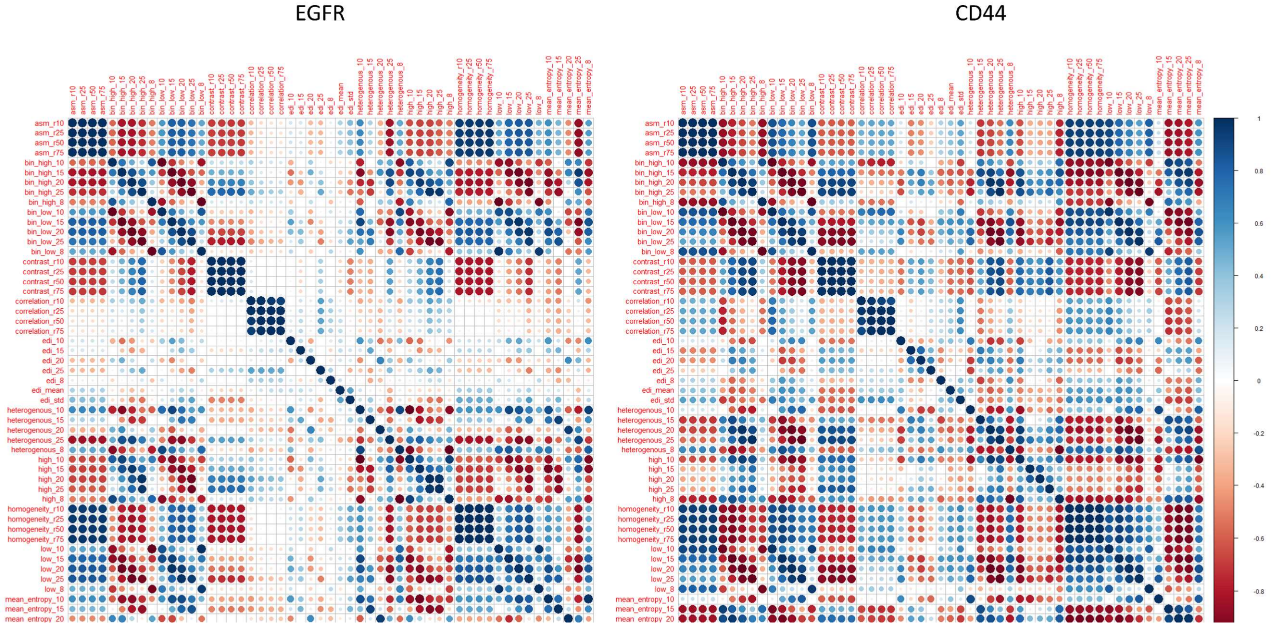

Figure S6 Correlation plots between spatial scores for different markers. *ASM* corresponds to angular second moment, *bin_high/bin_low* correspond to the ratio of homogenous-high/homogenous-low tiles without classification of heterogenous slides; *low/high/heterogenous* correspond to the tile ratios including heterogenous tiles, *edi_* corresponds to the EDI on entropy values, *edi_mean/edi_std* correspond to EDI on average/standard deviation of expression values per tile.

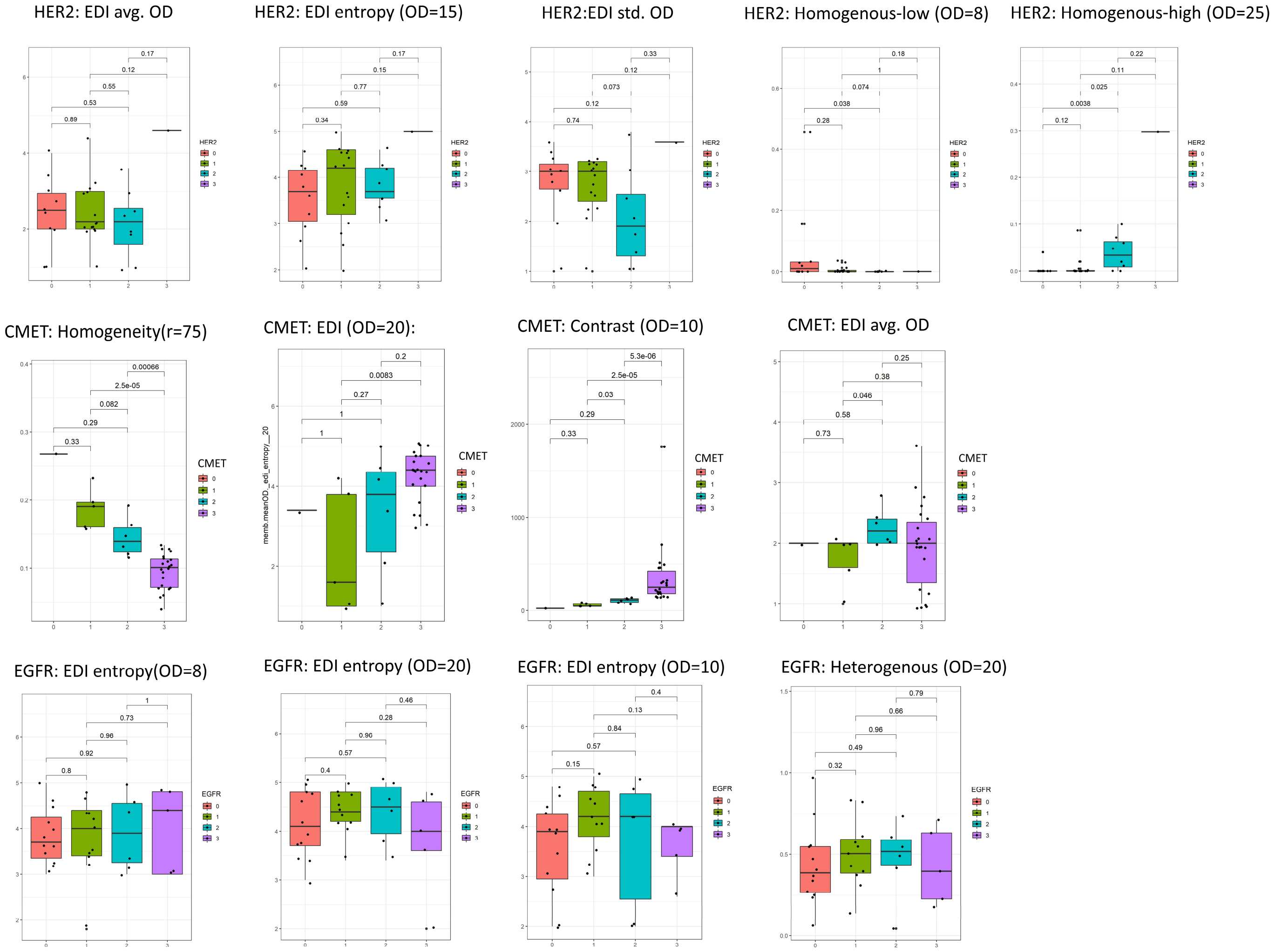

Figure S7 Concordance of spatial scores with pathologist scores.

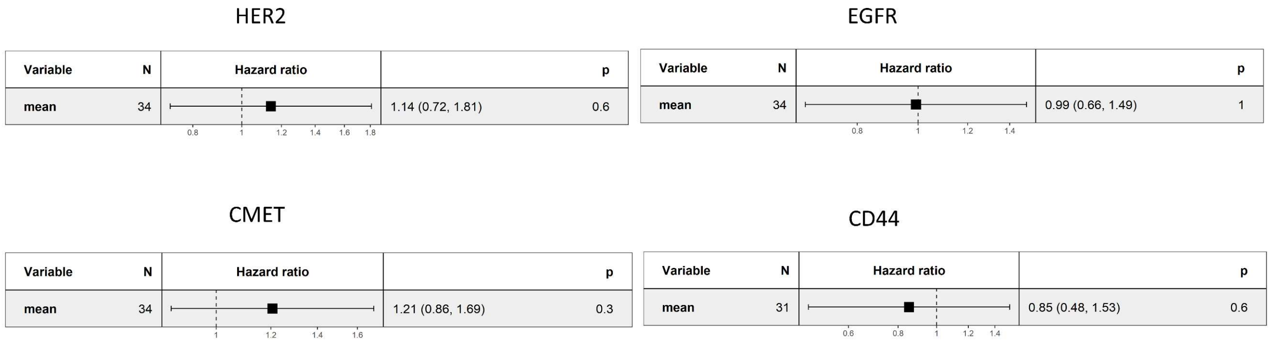

Figure S8 Cox regression for average marker expression.

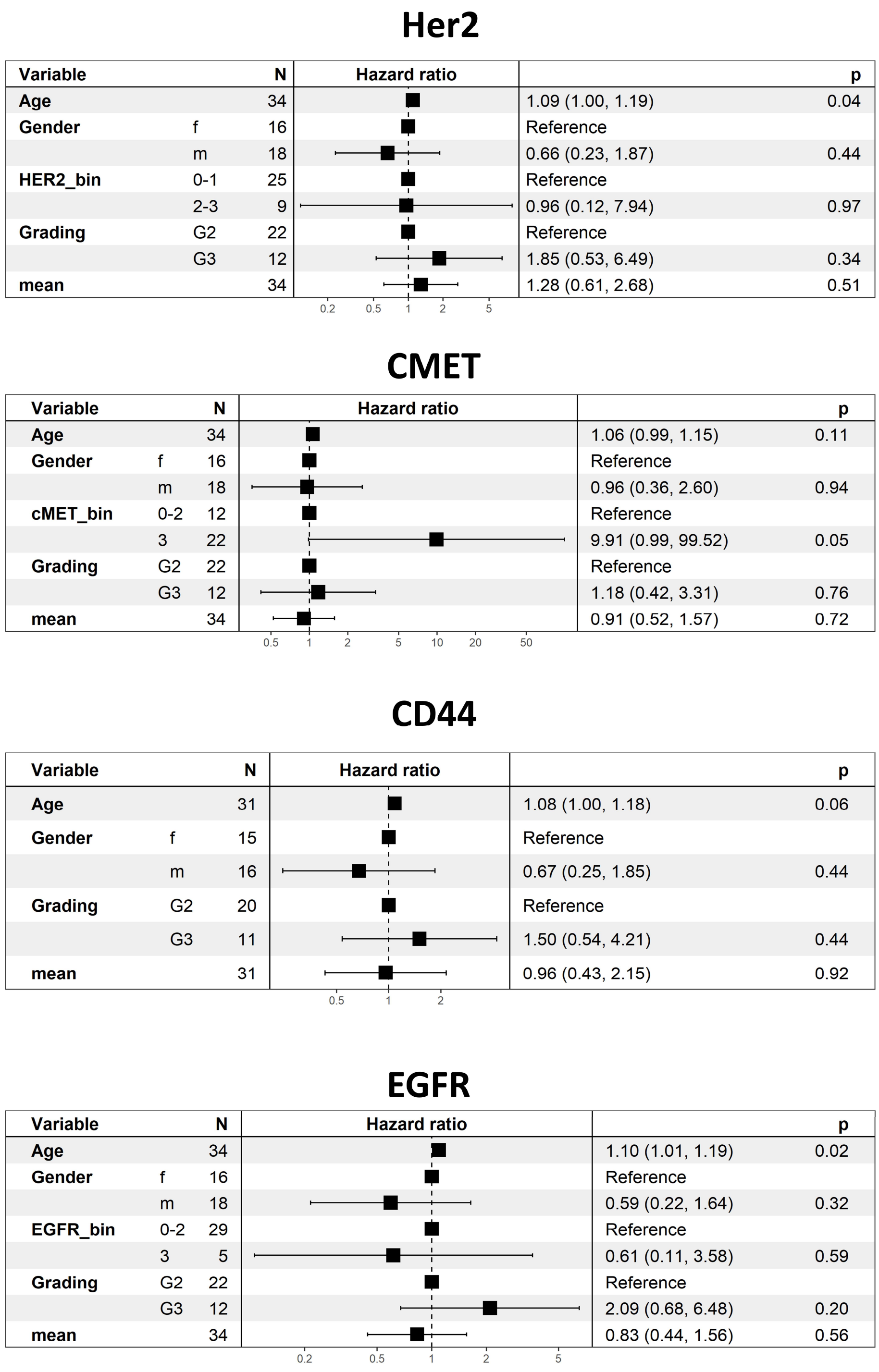

Figure S9 Base models for different marker combinations.

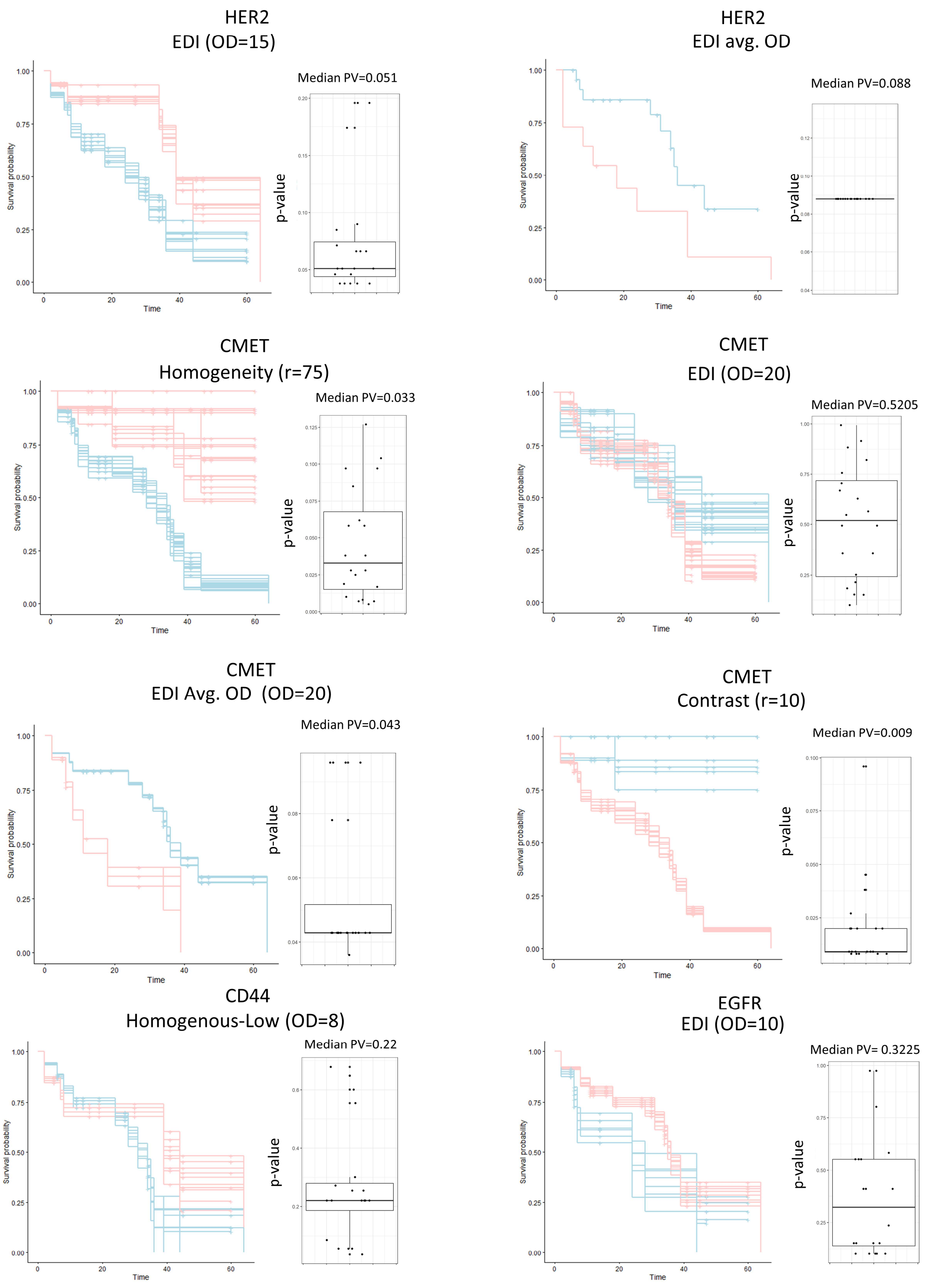

Figure S10 Repeated 20 x 10 fold cross validation results for spatial scores.

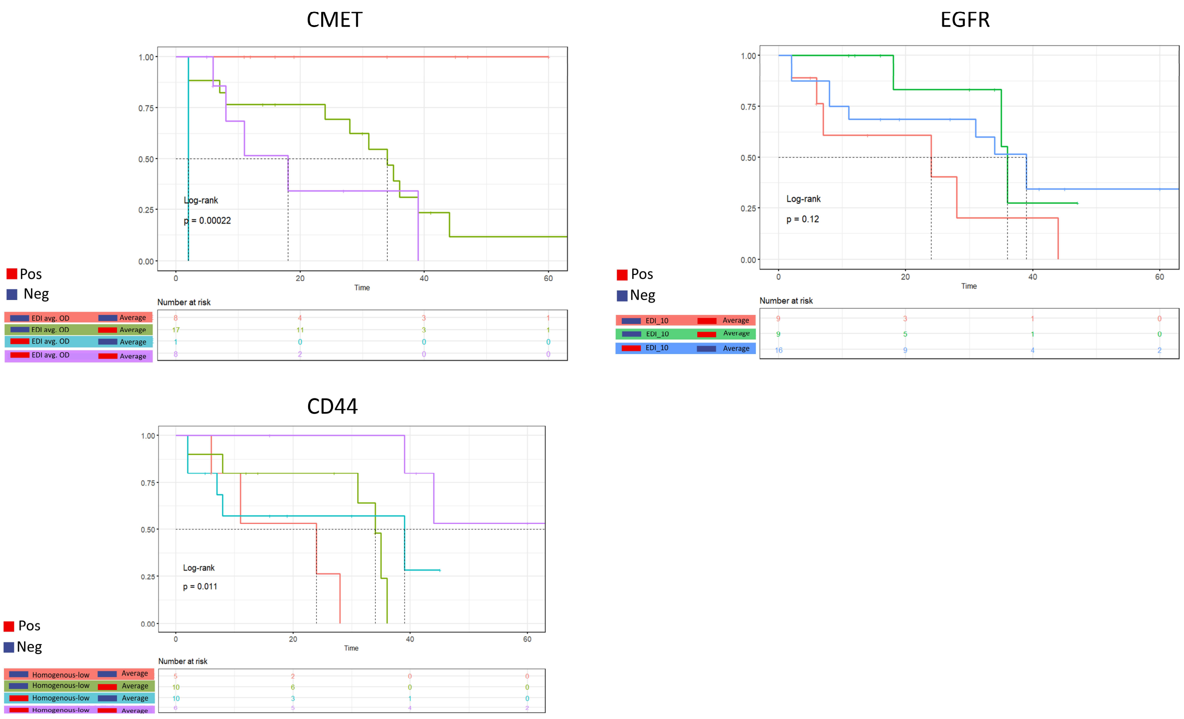

Figure S11 Patient stratification by combining spatial scores and average expression.
